# Health workforce cultural competency interventions: a systematic scoping review

**DOI:** 10.1186/s12913-018-3001-5

**Published:** 2018-04-02

**Authors:** Crystal Jongen, Janya McCalman, Roxanne Bainbridge

**Affiliations:** 1 School of Health, Medicine and Applied Sciences, Central Queensland University, Cnr Shields and Abbott Streets, Cairns, QLD 4870 Australia; 2 Centre for Indigenous Health Equity Research, Central Queensland University, Cnr Shields and Abbott Streets, Cairns, QLD 4870 Australia

**Keywords:** Cultural competence, Indigenous, Ethnic minorities, Cultural competence training, Health workforce development

## Abstract

**Background:**

Addressing health workforce cultural competence is a common approach to improving health service quality for culturally and ethnically diverse groups. Research evidence in this area is primarily focused on cultural competency training and its effects on practitioners’ knowledge, attitudes, skills and behaviour. While improvements in measures of healthcare practitioner cultural competency and other healthcare outcomes have been reported, there are concerns around evidence strength and quality. This scoping review reports on the intervention strategies, outcomes, and measures of included studies with the purpose of informing the implementation and evaluation of future interventions to improve health workforce cultural competence.

**Methods:**

This systematic scoping review was completed as part of a larger systematic literature search conducted on cultural competence intervention evaluations in health care in Canada, the United States, Australia and New Zealand published from 2006 to 2015. Overall, 64 studies on cultural competency interventions were found, with 16 aimed directly at the health workforce.

**Results:**

There was significant heterogeneity in workforce intervention strategies, measures and outcomes reported across studies making comparisons of intervention effects difficult. The two main workforce intervention strategies identified were cultural competency training and other professional development interventions including other training and mentoring. Positive outcomes were commonly reported for improved practitioner knowledge (9/16), skills (7/16), and attitudes/beliefs (5/16). Although health care (6/16) and health (2/16) outcomes were reported in some studies there was very limited evidence of positive intervention impacts. Only four studies utilised existing validated measurement tools to assess intervention outcomes.

**Conclusion:**

Training and development of the health workforce remain a principle strategy towards the goal of improved cultural competence in health services and systems. Diverse approaches are available to increase health workforce cultural competence. However, the effects of interventions beyond practitioner knowledge and attitudes remains unclear. Assessment of practitioner behavioural outcomes as well as measures of intervention impact on healthcare and health outcomes are needed to build a stronger evidence base.

**Electronic supplementary material:**

The online version of this article (10.1186/s12913-018-3001-5) contains supplementary material, which is available to authorized users.

## Background

While there is no doubt about the central role of culture in health and health care [1] the concepts of culture, cultural difference and cultural competence are complex and can be difficult to define [2]. Many varied definitions are used to describe cultural competence; one definition commonly used was provided by Cross, Bazron, Dennis and Isaacs (1989) [3]. Cross et al. define cultural competence as “a set of congruent behaviours, attitudes and policies that come together in a system, agency or among professionals that enable that system, agency or professions to work effectively in cross-cultural situations” (p. iv) [3]. This definition accounts for a range of intervention approaches which are used to improve the cultural competence of healthcare systems. One key approach to improving overall health care cultural competence is to develop the capacity of the health workforce to practice in a culturally competent manner.

Health professionals play a key role in determining the nature of interactions and patient experiences when accessing health care. Cultural and linguistic differences between healthcare providers and health service users can results in significant miscommunication [4], as well as service user mistrust [5], decreased satisfaction and disempowerment [6]. In contrast, practitioners’ increased cultural competence has been linked to increased patient satisfaction [7, 8], treatment adherence [9] and information seeking and sharing [8]. It is perhaps due to the key role that health practitioners play in determining the health care experiences of patients that improving health workforce cultural competency is one of the oldest and most predominant of cultural competence strategies [10, 11].

The general focus of cultural competence workforce interventions has been on educating and training the health workforce in the requisite and relevant knowledge, attitudes, and skills needed to effectively respond to sociocultural issues arising in clinical encounters [11]. Cultural competence training can include: understanding the central role of culture in all lives and how it shapes behaviour; respect and acceptance of cultural differences; learning to effectively utilise culturally adapted and culturally specific practices; and, continuous development of ones awareness of personal cultural influences and prejudices or biases [12–15]. Cultural competence training has mostly focused on developing knowledge, attitudes, awareness and sensitivity of those working in healthcare. However, the literature reiterates the need to reach further than this, and focus on teaching the skills needed to translate knowledge and awareness into tangible practitioner behaviours which can be consistently applied and assessed in healthcare encounters and settings [3, 10, 15, 16].

Different approaches to cultural competence training have been adopted over the years. Historically, there has been a greater focus on categorical approaches that involve teaching health providers information about particular cultural, ethnic or racial groups. Such approaches describe common health beliefs, attitudes and behaviours of particular groups and offer prescriptive advice about what to do and what not to do in clinical encounters [11]. Nevertheless, it has been acknowledged that categorical approaches are insufficient and problematic for numerous reasons.

To begin with, the categorical approach is critiqued for misrepresenting and oversimplifying the concept of culture as fixed and static [17] rather than a fluid and dynamic phenomenon in a process of constant change and adaptation [16, 18, 19]. Furthermore, the significant cultural, religious, ethnic and national diversity present in many countries means that it is not feasible to be familiar with all cultural perspectives practitioners may encounter [11, 18, 20]. Categorical approaches to cultural competence training may lead to stereotyping which can in fact increase cultural misunderstanding [11, 17, 20]. Such approaches have also been criticised for giving little attention to intra-group variability [19] and for failing to account for the ways in which acculturation and socioeconomic status effect different individuals ways of expressing and experiencing their culture [11].

Another key approach to cultural competence education and training which addresses some of the concerns identified with categorical approaches is the cross-cultural approach. A cross-cultural approach to cultural competence education and training is focused on teaching general knowledge, attitudes and skills relevant to navigating any cross-cultural situation [11, 18]. Some of these skills and attitudes were outlined by pioneers in cross-cultural medicine such as Berlin and Fowkes [21], Kleinman [22] and Leininger [23], and include: eliciting patients’ explanatory models of health issues and their causes; strategies for negotiating shared understanding and facilitating participatory decision-making in creating treatment plans; and understanding health and illness in its biopsychosocial context [18, 20]. As well as being applicable in clinical encounters with patients from varied cultural and ethnic backgrounds, such approaches have the advantage of being focused on specific skills that can be applied in healthcare encounters.

Cultural competence interventions have come to be considered a key strategy towards addressing racial and ethnic healthcare and health disparities that exist across Canada, Australia, New Zealand, the United States (hereto referred to as the CANZUS nations) [24]. For example, the release of *Unequal Treatment* in 2002 by the United States (U.S.) Institute of Medicine revealed the critical disparities in the quality of health care received by ethnic and racial minorities [25] and established cultural competence training for healthcare professionals as an important step in addressing these pervasive disparities [18, 26]. As a result, factors besides cultural differences and cultural barriers came to be included in the discourse and scope of cultural competence. These factors include patient mistrust of health practitioners and systems because of historical and contemporary experiences of discrimination and provider bias towards minority groups [20]. Cultural competence training can include developing an awareness of issues of gender, sexuality, and those such as racism, health practitioner and system bias and mistrust [18, 20]. Critical reflection on practitioner perspectives is also advocated. This includes critically reflecting on and acknowledging the limitations of “medico-centric” frameworks and the effects of dynamics of power and privilege associated with professional status [20].

Positive outcomes have been reported from cultural competency interventions targeting the health workforce, particularly for practitioner outcomes. In their literature review on educational interventions to improve the cultural competence of health care providers, Beach et al. [27] found excellent evidence of improved practitioner knowledge and good evidence of improved practitioner attitudes and skills . However, there is less evidence generally for the impacts of cultural competence education and training interventions on the patient healthcare and health impacts so crucial for determining broader intervention effectiveness. For example, in their review Beach et al. [27] found some evidence for effects of cultural competence education interventions on patient satisfaction. However, poor evidence was found for patient adherence and no health outcomes were reported. Lie et al. [28] reviewed cultural competency workforce interventions that included measures of health outcomes. Although seven studies were found, the studies were of low to moderate methodological quality and showed limited evidence of a positive relationship between cultural competency training initiatives and improved health outcomes.

This paper was developed as part of a broader review of cultural competency interventions in health care for Indigenous peoples and other minority ethnic/cultural groups in the CANZUS Nations [24], the findings of which have been published in our book [2]. The aim of the larger review was to assess the intervention strategies and measures used to increase cultural competence in health care in the CANZUS nations, along with the outcomes reported for these interventions. These countries were selected due to the commonalities in population health and colonial history that exist across these four settler countries [29, 30]. Several reviews have addressed common issues across the CANZUS nations, reporting particularly on Indigenous health issues [31, 32]. The larger review looks at various cultural competence interventions across multiple healthcare system levels or components. This paper is distinguished from this larger review in that it provides a detailed and evidence-based review of the findings specific to health workforce interventions, a discreet and unique approach to increasing cultural competence in health care.

In this article, we contribute to the existing literature base by providing a systematic scoping review of studies on workforce cultural competency education and training interventions from 2006 to 2015. Its purpose is to inform the implementation and evaluation of future interventions to improve the cultural competence of health professionals. In particular, this review aims to:

- Examine the definitions of culture, cultural difference and cultural competence adopted by the included studies;
- Examine the intervention strategies utilised by studies;
- Report on the measurement approaches taken to evaluate interventions;
- Examine the reported outcomes of included studies.

## Methods

As this review was completed as part of a larger systematic review, details on the inclusion/exclusion criteria, search strategy, identification, screening and inclusion of publications, as well as data extraction and analysis processes used in the broader systematic review have been reported elsewhere [2, 33, 34]. To summarise briefly, the review included peer-reviewed and grey literature published in English from January 1st 2006 to December 31st 2015. Included publications were those which evaluated an intervention designed to improve cultural competence in healthcare for Indigenous or other racial or ethnic minority groups in Australia, Canada, New Zealand or the United States (see Additional file 1 for an overview of the search strategy).

**Fig. 1:**
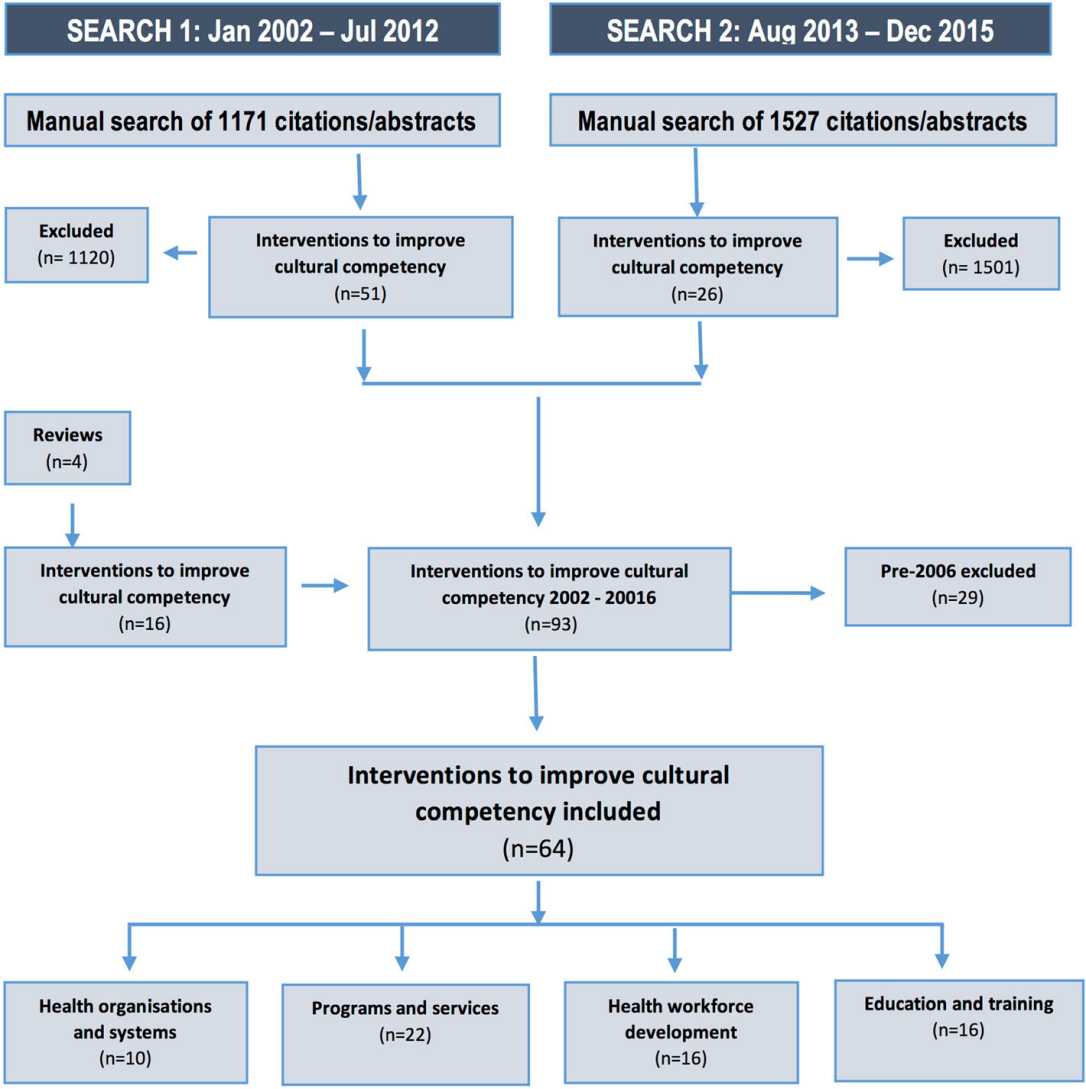
PRISMA search strategy flow chart

Our comprehensive, six step search strategy and blinded screening process resulted in 64 studies for inclusion in the review (see Fig. 1 for PRISMA search strategy flow chart). Data was extracted for all included studies (see Additional file 2) and the quality of included studies were assessed using the Effective Public Health Practice Project (EPHPP) [35] and Critical Appraisal Skills Programme (CASP) quality assessment tools [36].

**Fig. 2:**
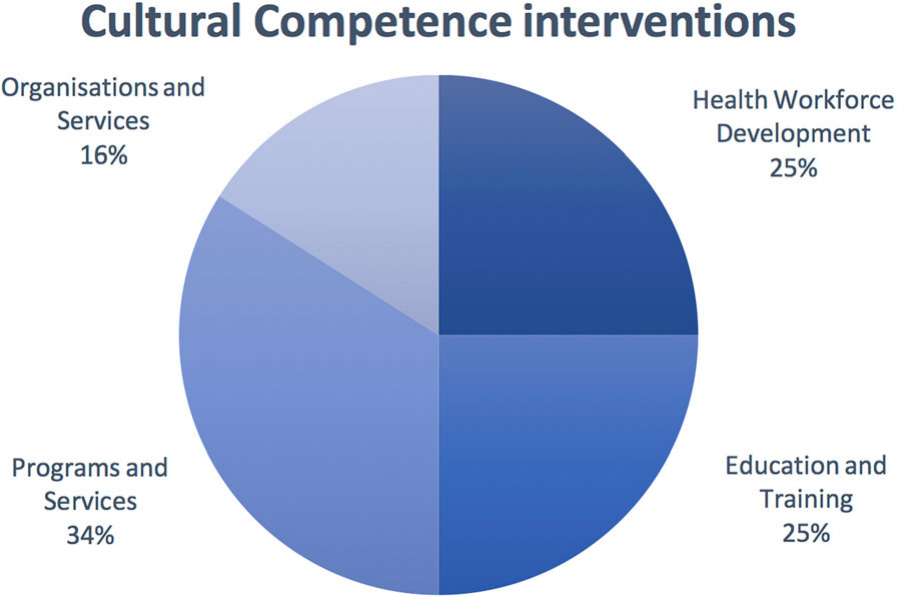
Cultural competence interventions by targeted healthcare level

The 64 studies found were organised according to the healthcare level or component being addressed. The authors identified four primary cultural competence intervention categories including: health workforce development (*n* = 16, 25%); student education and training (*n* = 16, 25%) [37]; programs and services (*n* = 22, 34%) [33], and; health organisations and systems (*n* = 10, 16%) [34] (see Fig. 2). The 16 evaluated interventions that aimed to increase cultural competency through health workforce development are reported in this paper.

## Results

### Definitions of culture, cultural difference and cultural competence

Across all included studies, none provided a definition of culture and only three provided a definition of cultural competence [38–40]. Although sharing some commonalities, all three definitions of cultural competence were distinct. There were also dissimilarities in the primary cultural differences discussed across studies which justified the need for cultural competence. Some included studies discussed cultural differences as distinctions in understandings of health that exist among different population groups, for example the holistic understandings of health held by Indigenous people and how this differs from mainstream approaches to health [41, 42]. Other studies discussed communication issues related to language discordance and cultural differences and how these affect clinical encounters, particularly for Hispanic populations in the United States [43–45].

### Intervention strategies

Across the included studies, diverse intervention strategies were used to address cultural competence at the workforce level. The two primary workforce strategies were cultural competency training interventions and professional development interventions aimed at improving the cultural competence of health services and practitioners. There was a significant variation in focus, content, mode of delivery and duration of interventions within these two primary strategies. There was also heterogeneity in the outcomes reported across the studies. The most common outcomes were for practitioner related cultural competence, along with some healthcare process and health outcomes. See Table 1 for a detailed summary of intervention strategies and outcomes of included studies.

**Table 1:** Summary of intervention strategies and outcomes

Publication	Aim	Intervention Strategies							Outcomes											
		Cultural Competency training			Professional Development		Delivery mode		Practitioner cultural competency outcomes					Healthcare/health outcomes				Other outcomes		
First Author year	Increased cultural competency	General	Specific	Minimal detail	Other training	Mentoring/Supervision	Multiple sites	Single sites	Knowledge	Attitudes/Beliefs	Skills	Behaviour	Confidence	Patient satisfaction	Patient trust	Practitioner satisfaction	Health outcomes	Research productivity	Training completion rates	Improved readiness to provide cc care
Aboriginal Workforce (2015) [50]	✓	x	✓	✓	x	x	✓	x	x	x	x	x	x	x	x	x	x	x	✓	x
Abbott (2014) [39]	~	x	x	x	x	✓	~	x	~	x	✓	✓	✓	x	x	x	x	x	x	x
Brathwaite (2006)	✓	✓	x	x	x	x	x	✓	✓	x	~	x	x	x	x	✓	x	x	x	x
Chapman (2014) [38]	✓	x	✓	✓	x	x	x	✓	✓	✓	x	x	x	x	x	x	x	x	x	x
Dingwall (2015) [42]	~	x	x	x	✓	x	✓	x	✓	x	✓	x	✓	x	x	x	x	x	x	x
Hinton (2012) [41]	~	x	x	x	✓	x	x	✓	✓	x	~	x	✓	x	x	✓	x	x	x	x
Khanna (2009) [46]	✓	✓	x	x	x	x	✓	x	✓	x	✓	x	x	x	x	x	x	x	x	x
Kutob (2009) [47]	✓	✓	~	x	x	x	✓	x	✓	x	✓	x	x	x	x	x	x	x	x	x
Liaw (2015) [52]	✓	x	~	~	✓	✓	✓	x	~	✓	✓	✓	x	x	x	✓	x	x	x	✓
Lopez-viets (2009)	~	x	x	x	x	✓	x	✓	x	x	x	✓	x	x	x	x	x	✓	x	x
McElmurry (2009) [45]	✓	x	✓	~	✓	x	✓	x	✓	✓	✓	~	x	x	x	~	✓	x	x	x
McGuire (2012) [44]	✓	x	✓	x	x	x	✓	x	✓	x	x	x	x	x	x	x	x	x	x	x
McRae (2008) [51]	✓	x	✓	✓	✓	x	✓	x	✓	✓	✓	x	✓	x	x	✓	x	x	x	x
Salman (2007) [48]	✓	✓	x	✓	✓	x	✓	x	~	✓	x	x	✓	x	x	~	x	x	x	x
Thom (2006) [49]	✓	✓	x	x	x	x	✓	x	x	x	~	✓	x	✓	✓	~	✓	x	x	x
Wu (2006) [43]	✓	x	✓	~	x	~	x	✓	x	x	x	x	x	✓	x	~	x	x	x	x

The symbol **✓** denotes evidence that the author(s) explicitly advanced adoption or support of the element of cultural competence, **~** denotes an implicit or inferred reference consistent with the intent of that element; and x denotes no evidence for that element

#### Cultural competence training

Eleven of the 16 studies reviewed (69%) provided cultural competence training to the health workforce as the primary intervention. The different approaches to cultural competence training and education discussed previously were reflected in the reviewed studies. Five reported interventions employed a cross-cultural approach focused on teaching broadly applicable knowledge and skills for cultural competence [40, 46–49]. Six interventions utilised a categorical approach, teaching practitioners about certain characteristics, beliefs and behaviours of relevant populations [38, 43–45, 50, 51]. Three studies using categorical approaches focused on Indigenous Australians [38, 50, 51], and three were specific to Latin American peoples [43–45]. Two cultural competency training interventions also included some degree of Spanish language training [43, 45]. See Table 2 for summaries of cultural competence workforce training approaches.

**Table 2:** Cultural competence training approaches

Publication	Training approach
Cross-cultural approaches	
Brathwaite (2006) [40]	The cultural competency training components of cultural awareness; cultural knowledge; cultural skill; cultural encounter and; cultural desire were addressed by teaching basic process of providing culturally competent care such as a) acknowledging intracultural diversity and the breadth and complexity of culture as something possessed by all; b) conducting cultural assessments of service users; c) learning from patients about their culture d) recognizing the processes of acculturation and cultural diversity within individuals; e) developing agreed upon treatment plans; and f) accommodating non-harmful health beliefs and practices which may differ from practitioners personal and professional culture.
Khanna (2009) [46]	The training program covered broad cultural competency topics such: as defining cultural and linguistic competency; understanding racial and ethnic health disparities; exploring the relationship between culture and health beliefs; and the importance of cultural competency in effective patient/provider communication. Following training, participants were expected to be able to: define culture and describe the spectrum of diversity; understand the differences between ethnicity, race and culture; recognize and define the concepts of intercultural and intracultural diversity; understand the difference between cultural generalizations and stereotypes; define and understand cultural competency; explain the concept of the cultural competency continuum, and; describe the use of explanatory models and their importance in the patient-practitioner interaction.
Kutob (2009) [47]	Learning objectives included things such as: distinguishing between the terms culture, ethnicity and race; utilizing the Ask, Share, Compare, Negotiate (ASCN) model with patients; describing evidence based information on health disparities as well as health beliefs and behaviours of Mexican American patients; managing potential barriers to blood sugar control for people with type 2 diabetes, and; the appropriate ordering of routine prevention services for diabetes patients. Participants were assessed for things such as their cultural self-awareness, their ability to be open and nonjudgmental, their avoidance of stereotyping, and ability to elicit patients’ explanatory model among others.
Salman (2007) [48]	Cultural workshop focused on understanding the components of cultural competence and its relevance to healthcare delivery.
Thom (2006) [49]	The competencies addressed were knowledge (such as knowledge of cultural identification and levels of agreement with respect to mainstream health beliefs), “communication skills (including listening, explaining, acknowledging, providing recommendations, and working effectively with interpreters); and cultural brokering (including negotiating a treatment plan with patient and family, understanding community resources available to patients, and working with then healthcare system to meet the needs of culturally diverse patients)”.
Categorical/Multicultural approaches	
Aboriginal Workforce (2015) [50]	Included an eLearning component providing knowledge on Aboriginal history, culture and people, and exploring key challenges to providing culturally appropriate care to Aboriginal people and communities and a face-to-face workshop component including both generic content (aimed at bridging what is learnt in the eLearning component) and local content (exploring the local communities being served).
Chapman (2014) [38]	Aimed to provide health practitioners with a comprehensive understanding of aspects of Aboriginal culture and ideology.
McElmurry (2009) [45]	Cultural workshops addressed Latino patients’ expectations of care and experiences with health services, the impacts of cultural beliefs and language barriers on issues in diagnosis, treatment, and popular herbal remedies frequently utilised in Latino cultures. In addition to cultural workshops, this intervention offered an intensive Spanish language course, or an integrated immersion program including Spanish language classes, cultural workshops, community-based clinical experiences and home-stays.
McGuire (2012) [44]	A training program focused on factors which may affect patient-practitioner communication and care such as: barriers in accessing health care in the U.S.; differences in health care systems in Latin America and the U.S.; expectations of Latino patients seeking care; social and cultural constructs of health and illness in Latino cultures, and; common health beliefs and practices such as the use of complementary medicine.
McRae (2008) [51]	Cultural awareness training workshop regarding working with Aboriginal Australians.
Wu (2006) [43]	Brief training introduced Latino cultural values and home remedies important to history taking, taught residents a few Spanish expressions to help establish rapport with Spanish-speaking patients, and discussed techniques for optimizing the use of interpreters in improving communication.

#### Professional development interventions

After cultural competence training, the next most common intervention type was professional development. Professional development interventions used both training and mentoring/supervision to increase the cultural competence of the health workforce. Five studies delivered training concentrated on particular health issues/fields or specific interventions [41, 42, 48, 51, 52], and four studies used mentoring/supervision strategies [39, 43, 52, 53]. Mentoring and supervision strategies aimed at increasing cultural competency were employed with individual practitioners, entire health services, and minority research faculty and students. See Table 3 for professional development intervention strategies.

**Table 3:** Professional development interventions

Publication	Other Training	Mentoring/Supervision
Abbott (2014) [39]		An intervention to explore (general practitioner) GP Supervisors and Medical Educators attention to cultural competency when providing supervision to Medical Registrars. Participants viewed a simulated consultation between an Aboriginal patient and GP Registrar highlighting inadequacies in communication and cultural awareness and documented teaching points to prioritize in supervision in response to the video consultation.
Dingwall (2015) [42]	Evaluated the effects of training in a culturally adapted Indigenous e-mental health application on Indigenous and non- Indigenous service providers’ awareness, perceived knowledge and confidence in using the app with Indigenous clients.	
Hinton (2012) [41]	Evaluated the effects of an Indigenous specific “Yarning about Mental Health” training for the Australian Drug and Alcohol workforce providing culturally appropriate strategies and tools for understanding mental health, promoting wellbeing, and delivering brief, evidence-based interventions.	
Liaw (2015) [52]	Participating medical practices partook in a cultural respect workshop which provided orientation to the ‘*Ways of Thinking Ways of Doing’* clinical re-design program designed to improve the cultural competency of General Practices.	Participating practices received support from a cultural mentor to guide the clinical re-design process.
Viets (2009) [53]		A university-based, culturally centred mentorship program which aimed to train and mentor junior faculty and graduate students from underrepresented backgrounds to conduct addictions-related research projects for Native American, Latino and rural communities and to develop culturally supported interventions (CSI) or adapted empirically supported interventions (ESI) for these communities.
McRae (2008) [51]	Pharmacists received training in culturally appropriate teaching strategies then delivered a culturally appropriate program designed to educate Aboriginal Health Workers about cardiovascular medicines management for Aboriginal people.	
Salman (2007) [48]	Alongside general cultural competency training, this intervention included training in ethno-geriatric care.	
Wu (2006) [43]		Individual cultural education sessions were provided to residents where language or cultural issues that emerged during specific clinical encounters were reviewed with the cultural educator.

#### Delivery mode

Another important distinction in the reviewed interventions was in the delivery mode chosen (Table 4 reports on interventions by setting, target group and delivery mode). Only 5/16 studies implemented an intervention in one practice setting. The majority of evaluated interventions (11/16, 69%) were implemented across multiple healthcare services. Studies reported on intervention delivered across multiple sites in one local area [45, 46, 48, 49, 52], targeting healthcare workers from diverse practice settings [39, 42, 51], or delivered state or nationwide [44, 47, 50]. Furthermore, there was such variance in the frequency and duration of cultural competency training interventions that an analysis of outcome effects related to course duration cannot be provided.

**Table 4:** Intervention setting, target group and delivery mode

Publication	Intervention setting and target group	Delivery mode
Aboriginal Workforce (2015) [50]	All health staff in a state based health system, encompassing primary and secondary health settings.	State-wide intervention delivered through a 2-h eLearning component and 6-h face-to-face workshop.
Abbott (2014) [39]	Training event for GP Supervisors (*n* = 71) and Medical Educators (*n* = 4).	Intervention delivered through two non-mandatory GP Supervisor training days involving practitioners from various practice sites.
Brathwaite (2006) [40]	Public Health Nurses (*n* = 75) employed at a Public Health Department.	The cultural competence program ran two-hour workshops over five consecutive weeks, with one booster session at 1 month. The intervention was delivered through one organisational site.
Chapman (2014) [38]	Emergency Department staff including nursing, clerical and allied health staff (*n* = 44) from in a hospital setting.	Three 2-h workshops delivered over 6 weeks to staff at one site.
Dingwall (2015) [42]	Training completed over one-year by health staff of diverse professions (*n* = 138) attending one of ten training courses held in various locations.	Multiple health professionals working across the state.
Hinton (2012) [41]	Training held for the Alcohol and Other Drug (AOD) workforce (*n* = 59), including AOD workers and counsellors, mental health and allied health workers.	Four 1-day training workshops held over a period of 2 years for members of two AOD workforce network agencies.
Khanna (2009) [46]	Healthcare providers and administrators (*n* = 60).	4 h long cultural competency training delivered to healthcare professionals working across two large regional medical groups.
Kutob (2009) [47]	Family Medicine Residents (*n* = 122).	A 1-h internet-based cultural competency course trialled on a national sample.
Liaw (2015) [52]	GP clinics (*n* = 10) and their staff.	Half-day cultural respect workshop, toolkit and cultural mentor to support a clinic re-design process implemented across numerous GP clinics in one region.
Lopez-Viets (2009)	Ethnic minority junior faculty members at a University (*n = 9*).	A four-year culturally centred mentorship program delivered in one University.
McElmurry (2009) [45]	Health care professionals and students (*n* = 386) across 5 ambulatory care sites.	A 3-year demonstration project offering training to health staff across a large regional health service network involving 5 healthcare sites. A six-session cultural workshop series and intensive Spanish language classes were offered as either an 8-week class series or 1–3-week integrated immersion program.
McGuire (2012) [45]	Health professionals (*n* = 63).	Education DVD delivered to healthcare professionals state-wide through conferences, community meetings and clinic training. A life nationwide webcast and satellite conference was also offered, and the training was accessible online. 26 states were represented in the webcast.
McRae (2008) [51]	Pharmacists (*n* = 12) and Aboriginal Health Workers (AHW) (*n* = 47).	Pharmacists and AHW’s based across 10 localities throughout a large regional area. Pharmacists attended an education weekend which included a 4-h cultural awareness session. The health worker education program occurred over 4 sessions ranging from 30 min to 1.5 h.
Salman (2007) [48]	Nursing staff (*n* = 202) in hospital settings.	Training was provided to nurses working across two major tertiary hospitals.
Thom (2006) [49]	Primary care physicians (*n* = 53) across diverse health care practice settings.	Intervention was implemented across four diverse health care practice sites. Three modules could be delivered as one half-day training or 3 separate sessions of 1–1.5 h.
Wu (2006) [43]	Medical residents delivering care to Spanish speaking parents (*n* = 250) in one large teaching hospital.	30-min group cultural workshop and two individual cultural mentoring sessions delivered to medical residents in one practice setting.

### Study quality

When assessing for study quality, ten of the 16 studies were found to be weak (63%), four moderate (25%) [38, 41, 47, 50] and two strong [43, 49].

### Intervention outcomes

The primary outcome measures reported in the reviewed cultural competency workforce development intervention evaluations were health practitioner knowledge (9/16) [38, 40–42, 44–47, 51], attitudes/beliefs (5/16) [38, 45, 48, 51, 52], skills (7/16) [39, 42, 45–47, 51, 52], behaviour (4/16) [39, 49, 52, 53], and confidence (5/16) [39, 41, 42, 48, 51]. Consistent with Beach et al. [27], our results show that many training interventions had positive outcomes independent of whether they were courses of longer or shorter duration. This finding was consistent whether training interventions used a cross-cultural approach to cultural competence training or a categorical approach, with positive outcomes reported across studies .

Although less common, healthcare outcomes such as practitioner satisfaction (4/16) [40, 41, 51, 52], patient satisfaction (2/16) [43, 49] and patient trust (1/16) [49] were evaluated in some studies. However, only one study reported improved healthcare outcomes in response to a cultural competency intervention, that of increased patient satisfaction [43]. Two studies evaluated the impacts of interventions on patient health outcomes [45, 49], but not significant improvements were reported. The limited evaluation on the health impacts of interventions in the reviewed studies means these results cannot contribute further to the relatively weak evidence-base on the impacts of cultural competence training on patient health outcomes demonstrated by previous research [28]. Three other outcomes reported were increased research productivity, training completion rates, and improved readiness to provide culturally competent care. See Tables 1 and 5 for a summary of outcomes by study.

**Table 5:** Study design, measures and outcomes

	Study Design and Measures	Outcomes
Abbott (2014) [39]	Content analysis to determine the type and detail of the planned feedback, field notes from workshop discussions and participant evaluations to gain insight into participant confidence in cross cultural supervision.	72% registrars referred to culture or to the patient’s Aboriginality; few (8%) documented plans to utilise national initiatives to support health care access for Aboriginal patients. A lack of supervisor confidence in providing guidance on cross-cultural consultation with Aboriginal patients was identified.
Aboriginal Workforce (2015) [50]	Data analysis of training completions to measure the percentage of health staff who completed training components; staff and participant interviews; and, web-based survey of Chief Executives.	Average of 35% of New South Wales (NSW) Health staff completed online training, with significant variation in completion of face-to-face component across Local Health Districts (LHD). Program implementation was found to be slower than anticipated.
Brathwaite (2006) [40]	Multiple time-series design to measure nurse cultural knowledge as measured by the Cultural Knowledge Scale (CKS).	Quantitative and qualitative data showed in an increase in participants cultural knowledge following the program.
Chapman (2014) [38]	Pre and post questionnaire to measure the cultural awareness (perceptions and attitudes) of staff.	Changes in staff perceptions, but not attitudes which remained neutral. A decrease in ambivalence.
Dingwall (2015) [42]	Pre and post questionnaire to measure participant knowledge and confidence in delivering e-mental health to Indigenous people.	Significantly improved perceived knowledge and confidence in using e-mental health tools with Indigenous clients after training.
Hinton (2012) [41]	Pre-post questionnaire to measure participant knowledge and skills.	Significant improvement in knowledge of the warning signs and treatment of mental illness and levels of confidence to assess, treat and communicate with Indigenous mental health clients.
Khanna (2009) [46]	Retrospective post- then pre- evaluation utilising a non-validated Cultural Competency Assessment (CCA) tool to measure changes in knowledge and skills related to the care of patients from diverse cultural and ethnic backgrounds.	Statistically significant change in participants self-reported knowledge and skills in providing culturally competent care.
Kutob (2009) [47]	RCT measuring changes in scores on the Cultural Competence Assessment Tool (CCAT), a self-assessment tool developed for the study.	Total CCAT scores significantly increased for experimental group participants (83.55 before the course to 192.09 after the course), but did not change for the control group.
Liaw (2015) [52]	Pragmatic pre- and post- evaluation using a practice site audit of cultural respect, health checks and risk factor management for Aboriginal patients in general practice. A Cultural Quotient (CQ) questionnaire was used to measure staff cultural strategic thinking, motivation and behaviour.	Practices improved their readiness to provide culturally appropriate care to Aboriginal patients; an increase in Aboriginal patients post intervention (*p* < 0.05).; and increase in cultural quotient score 74.8–89.8 (*p* < 0.05); and individual practice staff improved their cultural strategic thinking.
Lopez-Viets (2009)	Pre- and during intervention evaluation in measures of research productivity, including number of grant applications and awards, publications and professional presentations of mentees.	There was considerable increase in total mentee research productivity: a 200% increase in grant applications and awards, a 336% increase in publications, and a 144% increase in professional presentations.
McElmurry (2009) [45]	Qualitative written evaluations and pre- and post- program focus groups to measure participants experiences/perceptions, and haemoglobin A_1c_ (HbA_1c_) levels in patients.	Self-reported increased appreciation of cultural interpretations of health, increased knowledge and consideration of Latino health beliefs and practices, improved ability to interact with patients, and greater respect and appreciation for patients cultural views. Improvements in blood glucose control as measured by a drop in HbA_1c_.
McGuire (2012) [44]	Pre-post self-report survey measuring practitioner knowledge and confidence.	Significant (*p* < 0.001) improvements in knowledge and confidence.
McRae (2008) [51]	Repeated measures three-phase questionnaire and semi-structured, face-to-face, in-depth interview post-program to evaluate pharmacists confidence. A brief survey to measure acceptability of program to AHWs and an audit of attendance.	Significant improvements in confidence with Indigenous health issues and educating AHWs (*p* = 0.002); access to resources to deliver education (*p* = 0.005). Education program delivered to 80% of AHW’s in the region with positive reports of participant satisfaction.
Salman (2007) [48]	Pre-post questionnaire to measure practitioner self-reported cultural awareness and competence.	No effect sizes reported. Increases in proportion of participants rated as culturally aware and competent.
Thom (2006) [49]	Randomised Control Trial (RCT) measuring Patient-Reported Physician Cultural Competence (PRPCC) score, patient satisfaction with and trust in physician, and patient health outcomes of weight, blood pressure and glycosylated haemoglobin.	No significant improvement on any outcome measure for either intervention group. Lack of impact of physician training on health care provision.
Wu (2006) [43]	Comparative study with historical control measuring parent reported satisfaction with interpreter and healthcare experience.	Use of an in-person interpreter significantly increased Latino parents satisfaction (*p* < 0.001) versus phone interpreter, but a program using an interpreter to educate residents in cultural and language issues increased parents’ satisfaction more.

### Measures

There was significant diversity in the measurement tools used across studies to measure changes in cultural competence, with no studies using the same measurement tool. Seven studies measured practitioner cultural competence using a measurement instrument. Of these seven, four used validated measurement instruments [38, 40, 48, 52]. One study used a tool developed by selecting relevant items from existing, validated instruments (reporting a final alpha coefficients for subscales ranging from .70 to .97) [47], and another independently developed a Cultural Competency Assessment (CCA) tool [46]. Lastly, one study measured the knowledge of participants using a pre-post multiple choice questionnaire [44]. The two studies measuring patient satisfaction utilised previously established indicator tools [43, 49] (see Table 5 for further detail on measures).

## Discussion

One of the key issues across the cultural competence literature is the lack of consistent terminology and an agreed upon definition of cultural competence and related concepts [2, 54–56]. As part of this review, we were interested to examine the definitions of culture and cultural competence used across studies as well as the understandings of cultural difference used to justify the need for cultural competence. Across the included studies, there was a general lack of reporting on definitions of cultural competence, culture and cultural difference. The definitions of cultural competence that were provided in only three of the included studies were all different, confirming the lack of a consistent definition of cultural competence. The use of inconsistent terminology to describe approaches towards the goal of improving cultural competence seen across the literature was also seen in the included studies. Terms used included cultural awareness, cultural respect, cultural safety, cultural understanding, and culturally appropriate healthcare. Furthermore, cultural differences are complex and varied within and across different cultural and ethnic groups [2]. For this reason, it is only logical that the types of differences cultural competency approaches aim to address with be distinct for different population groups.

The interventions reported across the studies included in this review were varied. The primary intervention strategies used were cultural competence training and other professional development activities. Professional development activities included health issue/field or program-specific training, and mentoring/supervision approaches. These interventions represent a diversity of approaches taken to improve health workforce cultural competence across CANZUS nations. This diversity can be considered a strength, demonstrating the many opportunities available to facilitate the ongoing process of improving the cultural competence of the practicing health workforce. However, the heterogeneity of intervention approaches, measures and outcomes makes analysis of interventions and their outcomes more difficult. For this reason, we focus our discussion on the general trends that are seen across studies, particularly those using similar strategies to improve health workforce cultural competence.

Cultural competence training for the health workforce was the most frequently implemented intervention strategy reported across 69% of the included studies. Cultural competence training interventions were delivered to a range of health professionals. Although some studies reported on training delivered specifically to physicians or nurses, more commonly, cultural competence training was provided to a diverse range of healthcare professionals together. There were no apparent differences in the training delivered to specific or mixed healthcare professionals in the strategies or outcomes reported. This indicates that many cultural competence training interventions are quite generic in nature, and do not necessarily target specific skills and knowledge, or types of care relationships that exist in health care.

There were many commonalities across cultural competency training intervention strategies and outcomes. These commonalities help to shed light on some key strengths and limitations of common approaches to cultural competence training. For example, out of the eleven studies evaluating cultural competency training interventions, six utilised a categorical approach and five implemented a cross-cultural approach. Interventions using either categorical or cross-cultural approaches reported positive outcomes around practitioner knowledge, attitudes/beliefs and reported skills and confidence. Due to the heterogeneity in measurement instruments and assessment methods, we were unable to discern whether either of these approaches to cultural competence training had a greater impact on particular learning outcomes. Despite this, there are some important issues pertaining to these approaches.

As discussed in the introduction, categorical approaches to cultural competence training can run the risk of increasing cultural misunderstanding if they do not account for inter-group variability. There are however certain instances in which categorical cultural competence training approaches can be effective or appropriate [18]. For example, if the cultural competence training is teaching about cultures of local-level populations with the help of local cultural experts, this can help to build cultural competence. Two of the included studies using categorical approaches included a focus on local level populations in line with this recommendation [44, 50]. The three remaining categorical cultural competence training studies either did not utilise such an approach, or did not report it. Another instance in which categorical approaches may be appropriate is when knowledge which has a clear, evidence-based effect on health care delivery or patient outcomes is being taught. Only one study utilising a cross-cultural approach to cultural competence training mentioned teaching such evidence-based knowledge [47]. Aside from these instances, to avoid generalisations which may lead to cultural misunderstanding, a more suitable tactic is to learn as much as possible directly from patients about their own sociocultural perspectives and how they see this impacting their encounters with healthcare practitioners [18].

Processes and skills for learning directly from patients is something that is commonly addressed in cross-cultural education models which have been established to inform the training of health professionals in culturally competent care [57]. For example, Kleinman’s explanatory model of disease [22] is a tool which can be used to facilitate cross-cultural communication, increasing understanding between patients and providers by eliciting patients own explanation of their health and or/illness. This tool is designed to help health providers better understand people’s health beliefs, personal and social meanings attached to health issues, and expectations about the therapeutic process [22]. Another key model for cross-cultural education is the LEARN (Listen, Explain, Acknowledge, Recommend, Negotiate) model developed by Berlin and Fowkes (1983) [21]. The LEARN model focuses on teaching generic skills for communication and negotiation that can be applied across all interactions when negotiating difference (cultural or otherwise) in the patient-practitioner encounter. Yet despite the existence and use of these models for many years, only two studies reviewed [47, 49] identified utilisation of both the LEARN model and Kleinman’s explanatory model.

To establish the relative impacts of different approaches to cultural competence training, comparative evaluations of interventions are needed to assess impacts using the same measurement instruments. Given the level of heterogeneity in cultural competency training interventions, a tool to assess the themes, concepts, methods and learning objectives of training interventions, such as the one utilised by Dolhun, Muñoz and Grumbach [58] would contribute greatly towards the comparison of outcomes between interventions. To facilitate greater analysis and comparison of cultural competency training approaches, it is important that evaluations provide sufficient detail on training approaches and content. This kind of detail was something that was lacking in many of the cultural competence training studies reviewed. Four of the cultural competence training studies using categorical approaches [38, 43, 50, 51] and one using a cross-cultural approach [48] did not provide sufficient information to clearly ascertain the content and focus of the training.

Despite the importance of issues of racism, discrimination and practitioner bias as issues to be addressed in culturally competent health care, none of the reviewed studies evaluating cultural competence training interventions explicitly discussed issues of racism and practitioner bias or stated that these issues were addressed in cultural competence training. Only one intervention evaluated addressed distinguishing between cultural generalisations and stereotypes in a cultural competence training program [46]. The lack of attention to issues of racism and bias is consistent with the findings of other cultural competence reviews. Beach et al. [27], for example, found only two of 34 studies which mentioned concepts of bias, racism or discrimination . Truong et al. (2014) found that although these issues were noted in some cultural competence literature reviews, they were not addressed as outcomes measures [54]. Considering the impact of issues such as racism and practitioner bias on healthcare disparities [2, 25, 59] interventions to improve health workforce cultural competence should address these issues and include them in outcome evaluations.

This review found two types of professional development strategies reported in included studies that have not been commonly reported in cultural competence literature reviews. One such strategy was training interventions other than cultural competence training which also aimed to increase healthcare workforce cultural competence. These training approaches included training regarding specific health issues/fields (eg. Indigenous mental health and wellbeing [41] and ethno-geriatric care [48]) and training in particular service-level interventions (eg. A culturally adapted Indigenous e-mental health mobile phone application [42], culturally appropriate teaching strategies for training Aboriginal Health Workers [51], and orientation to a cultural respect clinical re-design program [52]). It is recommended that a whole of organisation approach is taken where efforts to improve cultural competence are integrated into all professional development endeavours within a healthcare service [60]. These types of health issue or program specific training interventions are one strategy towards this goal. These training interventions demonstrate the different ways in which efforts to increase health professional cultural competence can be integrated into diverse professional development initiatives.

Mentoring and supervision was another strategy found [39, 43, 52, 53], with studies demonstrating the versatility and potential of this approach as a cultural competency workforce development strategy. Mentoring is a common and effective approach towards personal and career development in the workplace [61]. Mentoring relationships are focused on mentee’s learning and encourage a reflective dynamic where openness to feedback is embraced [62]; hence mentoring strategies could encourage the kind of life-long learning processes needed to continuously strive towards cultural competence. A significant focus of literature on mentoring and supervision in the context of cultural competence in the health workforce has been regarding supervision for minority practitioners by Caucasian supervisors [62–66]. However, there are also examples of research which explores the value of cultural mentoring in promoting the cultural competence of doctors working with Aboriginal and Torres Strait Islanders [67]. The potential of mentoring and supervision approaches to improve health practitioner cultural competence is a a research area worth further exploration and testing for its efficacy and impact.

This review did not purposely search for intervention studies aiming to improve health practitioner linguistic competence. However, considering the evidence demonstrating the impact of language discordance on patient satisfaction and quality of care [68, 69], the relative absence of efforts to address linguistic differences in most cultural competence workforce development interventions is discouraging. One of the reviewed studies provided Spanish language courses and an immersion program for health practitioners [45]. Another intervention focused on evaluating the effectiveness of interpreter services taught medical residents the use of Spanish expressions within a cultural education training program [43]. This lack of training to address language discordance in cultural competence training is consistent with previous research. Dolhun, Muñoz and Grumbach (2003) found that medical schools rarely addressed language issues in cultural competency course content, such as through teaching about the use of interpreters [58]. Interventions to improve cultural competence in the health workforce in CANZUS nations ought to address linguistic competence as a core aspect of cultural competence, particularly for populations who do not speak English as a first language.

The included studies report some positive effects of workforce cultural competence interventions, particularly on health professionals’ knowledge, skills and attitudes or beliefs. However, they did not report on behavioural outcomes of cultural competence interventions. One study assessed patient-reported physician cultural competence behaviours but found no changes following the intervention [49]. The only behavioural changes reported in studies included increased research productivity [53] and physical changes in practice settings to be more culturally sensitive [52]. Hence, the majority of cultural competence training for the health workforce remains focused on building awareness and associated changes in attitudes [60]. However, knowledge and attitude-based outcomes are not sufficient to demonstrate practitioner cultural competence. In order to build a stronger evidence base on the impact of cultural competency workforce interventions it is important that evaluations include assessment of practitioner behavioural outcomes. Cultural competence training approaches should prioritise the teaching of practical skills and the application of these skills in practice, as well as their assessment through demonstrable practitioner behaviour [12, 16, 57]. Assessment of behavioural outcomes could also contribute to evaluation of workforce cultural competence training impacts on patient healthcare and health outcomes. The assessment of healthcare and health outcomes are very important if we hope to demonstrate that cultural competence interventions do in fact impact on the healthcare disparities so frequently used to justify cultural competence interventions [2]. However, only two of the reviewed studies reported healthcare outcomes with one reporting improvements in patient satisfaction [43] and the other reporting no effect [49]. Health outcomes were assessed in two studies [45, 49] however neither reported significant changes as a result of interventions impacts. As long ago as 2003, Betancourt outlined a potential approach to cultural competence education and training intervention evaluation which assesses behavioural outcomes related to knowledge and skills taught and their impact on healthcare and health outcomes [18]. Cultural competence training and other workforce development interventions would greatly benefit from applying such an evaluation approach.

There are several key issues in the measurement and evaluation of cultural competence training and workforce development interventions which have been identified in previous literature and are mirrored in the studies reviewed. One concern is the lack of consistency in measurement instruments used to assess intervention outcomes, especially among cultural competency training evaluations. Similar to previous research [27, 70] we found no studies using the same assessment tool and little uniformity across studies in measurement of outcomes, even within the same outcome categories. This lack of consistency in measurement tools makes it difficult to compare intervention outcomes and effectiveness across studies.

The over-reliance of self-report measures is an ongoing limitation and concern across the cultural competency literature [14, 54, 70]. Self-report measures were the most common method of evaluation, utilised in 69% of included studies. However self-report measures are highly subjective and cannot be seen as predictive of resulting behaviour in clinical encounters [18]. Due to the effects of social-desirability bias, participants might select responses seen as socially appropriate but which are not reflective of their true beliefs [18, 70, 71]. To improve the evidence supporting their effectiveness, interventions aimed at improving health practitioner cultural competence need to move beyond the reliance on self-assessmen measures [54].

Patient assessed practitioner cultural competence is one potential approach to evaluating the impact of cultural competency training interventions which could be used instead of, or in addition to, practitioner self-assessment. Patient assessed practitioner cultural competence has been associated with improved healthcare and health outcomes [72, 73], however there is less evidence linking patient-assessed practitioner cultural competence and associated positive outcomes to impacts of cultural competence training. In the reviewed studies, only one assessed patient reported physician cultural competence behaviours and its correlation with patient satisfaction and trust with no impacts reported [49]. To increase the objectivity of the evidence base for the impact of cultural competency interventions, consistent assessment of patient perceived practitioner cultural competence, as well as healthcare and health outcomes, are needed.

### Limitations

The publications reviewed were identified using a search strategy including electronic databases, websites/clearinghouses and reference lists of reviews designed to discover peer and non-peer reviewed publications that evaluated health service cultural competency interventions. Therefore, it is highly likely that the studies in this review are representative of published cultural competence research from CANZUS nations. However, being a non-exhaustive search strategy it is possible some relevant publications were not found. Furthermore, considering the relative paucity of published studies found in this search, despite the inclusion of studies across four countries in a 10 year time-frame, the few studies found may suggest that many interventions are either not evaluated or not published. The reliance on published peer-reviewed and grey literature is therefore another limitation of this review.

The heterogeneity of included research aims, interventions and outcomes in the included studies is a further limitation. Although this heterogeneity is useful for demonstrating the diversity of approaches which can be taken to improve cultural competence in healthcare, it makes it difficult to draw firm conclusions about the nature of interventions and their associated outcomes. Additionally, because of the breadth and complexity of cultural competence, this review only included studies which explicitly addressed improving cultural competence as an aim of interventions; this possibly excluded studies which implicitly aimed to increase cultural competence. For example, our search did not produce any studies on the recruitment and retention of minority staff as a workforce development, cultural competence strategy.

## Conclusion

The studies which informed this review demonstrate a great diversity in approaches taken to address the cultural competence of the health workforce. Research exploring the comparative benefits of different approaches to cultural competence training as well as the benefits of other professional development opportunities such as mentoring and supervision would be of value to advance knowledge in this area. Although several positive outcomes were reported across the included studies, consistent evaluation approaches are needed to build the evidence base on intervention impacts. In particular, greater focus is needed on evaluating the application of knowledge, attitudes and skills in practice and the impacts of cultural competence interventions on specific practitioner behaviours and their subsequent impact on healthcare and health outcomes.

## Additional files

Additional file 1: Searches 1 and 2. Search summary for searches 1 and 2. This figure provides a summary of the literature searches undertaken to inform this review. The search summary details the peer-reviewed electronic databases as well as websites and clearinghouses searches, along with the search terms used. (JPEG 1745 kb)

Additional file 2: Data extraction table. Cultural competence workforce interventions data extraction table. This table provides detail on the data extracted from reviewed studies including: the author, year and publication type; the country and population; participants and health care setting; intervention type; study design; outcome measure or indicator; assessed outcomes; and study quality. (DOCX 108 kb)

## Abbreviations

AHW: Aboriginal health workers
AOD: Alcohol and other drug
ASCN: Ask, share, compare, negotiate
CANZUS: Canada, Australia, New Zealand, United States
CASP: Critical appraisal skills programme
CCA: Cultural competency assessment
CCAT: Cultural competence assessment Tool
CKS: Cultural knowledge scale
CQ: Cultural quotient
CQU: Central Queensland University
CSI: Culturally supported interventions
EPHPP: Effective public health practice project
ESI: Empirically supported interventions
GP: General practitioner
HbA_1c_: Hemoglobin A_1c_
LEARN: Listen, explain, acknowledge, recommend, negotiate
LHD: Local health districts
NSW: New South Wales
PRISMA: Preferred reporting items for systematic reviews and meta-analyses
PRPCC: Patient-reported physician cultural competence
RCT: Randomised control trial
U.S.: United States
