# Supplementary material for: Health workforce cultural competency interventions: a systematic scoping review
Source: BMC Health Serv Res. 2018 Apr 2;18:232. doi: 10.1186/s12913-018-3001-5 (PMC5879833; doi:10.1186/s12913-018-3001-5)
Supplement: Supplementary file 2 — Data extraction table. Cultural competence workforce interventions data extraction table. This table provides detail on the data extracted from reviewed studies including: the author, year and publication type; the country and population; participants and health care setting; intervention type; study design; outcome measure or indicator; assessed outcomes; and study quality. (DOCX 108 kb) [file 12913_2018_3001_MOESM2_ESM.docx]

Supplementary file 1: Cultural competence workforce interventions data extraction table

| **Author, year and publication type** | **Country and population** | **Participants and Health care setting** | **Intervention type** | **Study design** | **Outcome measure or indicator** | **Outcomes assessed** | **Study quality** |
| --- | --- | --- | --- | --- | --- | --- | --- |
| **Abbott, et al. (2014)**  Journal article | Australian  GP supervisors and Medical Educators | 64 participants  Training event for GP supervisors and Medical Educators | A simulated consultation between a GP Registrar and an Aboriginal patient, illustrating inadequacies in communication and cultural awareness, was viewed by participants. Participants documented teaching points to prioritise in supervision arising from the consultation | Content analysis to determine the type and detail of the planned feedback, field notes from workshop discussions and participant evaluations to gain insight into participant confidence in cross cultural supervision | n/a | 72% registrars referred to culture or to the patient's Aboriginality; few (8%) documented plans to utilise national initiatives to support health care access for Aboriginal patients. A lack of supervisor confidence in providing guidance on cross-cultural consultation with Aboriginal patients was identified | Weak |
| **Aboriginal Workforce (2015)**  Evaluation Report | Australia  NSW Health staff | All health staff across state-government health services | State-wide Aboriginal cultural training framework delivered via a 2-hour e-learning and six-hour face-to-face workshop | Mixed-methods: Data analysis of training completions; Staff and participant interviews; web-based survey of Chief Executives | Percentage of health staff who have completed training components. | Average of 35% of NSW Health staff completed online training, with significant variation in completion of face-to-face component across LHD’s | Moderate |
| **Braithwaite, et al. (2006)**  Journal article | Canada  Public Health Nurses | 76 public health nurses working in Public Health Department | Cultural competence educational programme to increase Public Health Nurses’ cultural knowledge | Multiple time-series design with quantitative and qualitative data | Nurse cultural knowledge as measured by the Cultural Knowledge Scale (CKS) | Quantitative and qualitative data showed in an increase in participants cultural knowledge following the program | Weak |
| **Chapman, et al. (2014)**  Journal Paper | Australia  Nursing, clerical and allied health staff | 44 Emergency department staff | Aboriginal and Torres Strait Islander cultural awareness training | Pre and post questionnaire | Cultural awareness (perceptions and attitudes) of staff | Changes in staff perceptions, but not attitudes which remained neutral. Decrease in ambivalence | Moderate |
| **Dingwall, et al. (2015)**  Journal Paper | Australia  Health service providers working with Indigenous people | 130 participants from various services across a state | Indigenous e-mental health training course | Pre and post questionnaire | Participant knowledge and confidence in delivering e-mental health to Indigenous people | Significantly improved perceived knowledge and confidence in using e-mental health tools with Indigenous clients after training | Weak |
| **Hinton, et al. (2012)**  Journal Paper | Australia  Alcohol and Other Drug (AOD) staff | 59 participants from the Alcohol and Other Drug (AOD) workforce | Culturally adapted Indigenous Mental Health and Wellbeing training workshop for the AOD workforce | Pre-post questionnaire | Knowledge and skills | Significant improvement in confidence and knowledge related to Indigenous mental health and wellbeing, including; improved knowledge of the warning signs and treatment of mental illness; in levels of confidence to assess, treat and communicate with Indigenous mental health clients | Moderate |
| **Khanna, et al. (2009)**  Journal Paper | U.S.  Health-care providers and administrators | 43 participants from regional medical groups | 4-hour cultural competency workshop | Retrospective post- then pre- evaluation  29-item Cultural Competency Assessment (CCA) tool – not validated | Changes in knowledge and skills related to the care of patients from diverse cultural and ethnic backgrounds | Statistically significant change in participants self-reported knowledge and skills in providing culturally competent care | Weak |
| **Kutob, et al. (2009)**  Journal Paper | U.S.  Family medicine residents | 122 participants from a random national sample | A skills-focused, internet-based course on cultural competence in the context of type-2 diabetes | RCT | Changes in scores on the Cultural Competence Assessment Tool (CCAT), a self-assessment tool developed for the study | Total CCAT scores significantly increased for experimental group participants (83.55 before the course to 192.09 after the course), but did not change for the control group | Moderate |
| **Liaw, et al. (2015)**  Journal Paper | Australia  Mainstream General Practices | 14 staff from 10 different GP practices | Cultural respect, whole-of-practice clinical re-design program for GP practices | Pragmatic pre- and post- qualitative and quantitative | Cultural respect, health checks and risk factor management for Aboriginal patients in general practice | Increase in Aboriginal patients post intervention (p <0.05); Increase in cultural quotient score 74.8 - 89.8 (p<0.05); Practices improved readiness to provide culturally appropriate care to Aboriginal patients; Individual practice staff improved their cultural strategic thinking. | Weak |
| **Lopez Viets, et al. (2009)**  Journal Paper | U.S.  Ethnic minority faculty members | 9 mentees and 3 mentors in a University setting | A culturally centred mentorship program for ethnic minority faculty | Pre-intervention and during intervention evaluation | Research productivity, number of grant applications and awards, publications and professional presentations of mentees. | There was considerable increase in total mentee research productivity: a 200% increase in grant applications and awards, a 336% increase in publications, and a 144% increase in professional presentations. | Weak |
| **McElmurry, et al. (2009)**  Journal Paper | United States  Health professionals and students | 386 participants across 5 Ambulatory care sites in a health service network | Cultural workshops and an intensive Spanish language course, or an integrated immersion program including Spanish language classes, cultural workshops, community-based clinical experiences and home-stays. | Qualitative written evaluations and pre- and post- program focus groups | Participants experiences/perceptions, and haemoglobin A_1c_ (HbA_1c_) levels in patients. | Self-reported increased appreciation of cultural interpretations of health, increased knowledge and consideration of Latino health beliefs and practices, improved ability to interact with patients, and greater respect and appreciation for patients cultural views. Improvements in blood glucose control as measured by a drop in HbA_1c_, | Weak |
| **McGuire, et al. (2012)**  Journal paper | U.S.  Healthcare professionals | 515 health professionals delivered state and nation wide | Educational DVD on Latino immigrants | Pre-post self-report survey | Practitioner knowledge and confidence | Significant (p<0.001) improvements in knowledge and confidence. | Weak |
| **McRae, et al. (2008)**  Journal Paper | Australia  Pharmacists and Aboriginal Health Workers (AHWs) | 12 Pharmacists and 47 AHW’s across a regional area | A culturally appropriate, pharmacist-led cardiovascular medicines education program for AHWs | Post-test with repeated measures | Confidence of pharmacists and acceptability of program to AHWs | Significant improvements in confidence with Indigenous health issues and educating AHWs (p = 0.002); access to resources to deliver education (p = 0.005). Education program delivered to 80% of AHW’s in the region with positive reports of participant satisfaction | Weak |
| **Salman, et al. (2007)**  Journal paper | U.S.  Nursing staff | 207 Geriatric Nurses in hospital setting | Geriatric care training program: cultural workshop and ethno-geriatric education | Pre-post questionnaire | Practitioner self-reported cultural awareness and competence | No effect sizes reported. Increases in proportion of participants rated as culturally aware and competent | Weak |
| **Thom, et al. (2006)**  Journal Paper | U.S.  Primary care physicians | 53 Primary Care Physicians and 429 patients across various healthcare practice setttings | Feedback on patient perceived cultural competency and patient satisfaction and trust delivered to all participants. Cultural competency training provided to experimental sites | RCT | Patient perceptions of physician cultural competence  Patient satisfaction with and trust in physician  Patient health outcomes | No significant improvement on any outcome measure.  Lack of impact of physician training on health care provision. | Strong |
| **Wu, et al. (2006)**  Journal Paper | U.S.  Medical residents and interpreters for Spanish-speaking patients | 49 Paediatric residents in a large teaching hospital  250 limited English proficient Spanish-speaking parents | Cultural education program and one-on-one mentoring regarding language and culture issues | Comparative with historical controls | Parent reported satisfaction with interpreter and healthcare experience | Use of an in person interpreter significantly increased Latino parents satisfaction (p<0.001) versus phone interpreter.; but a program using an interpreter to educate residents in cultural and language issues increased parents’ satisfaction more. | Strong |
